# A depositional taphonomy model for sedaDNA based on biotic profiles of contrasting sedimentation regimes

**DOI:** 10.1093/pnasnexus/pgag271

**Published:** 2026-08-10

**Authors:** Teri A Hansford, Vivian Higgs, Vincent Gaffney, Logan Kistler, Robin G Allaby

**Affiliations:** School of Life Sciences, Gibbet Hill Campus, University of Warwick, Coventry, Warwickshire CV4 7AL, United Kingdom; School of Life Sciences, Gibbet Hill Campus, University of Warwick, Coventry, Warwickshire CV4 7AL, United Kingdom; School of Archaeological and Forensic Sciences, University of Bradford, Bradford, Yorkshire BD7 1DP, United Kingdom; Department of Anthropology, National Museum of Natural History, Smithsonian Institution, Washington, DC 20560, USA; School of Life Sciences, Gibbet Hill Campus, University of Warwick, Coventry, Warwickshire CV4 7AL, United Kingdom

## Abstract

Sedimentary ancient DNA (sedaDNA) has become an important tool in Quaternary science, but still little is understood of its taphonomy and whether sedaDNA represents the local vicinity or originates from distant sources. Here, we show key insights can be made about the physical origins of sedaDNA by integrating sedimentological and sedaDNA data into a sediment influx depositional model. We reconstruct contrasting taphonomic regimes of lacustrine, alluvial, terrestrial, and permafrost systems. Lacustrine systems show signals of regional catchment sources of sedaDNA, while alluvial, terrestrial, and permafrost systems show a greater influence of DNA from the immediate local environment. However, there is a prevalence of mixed ecosystem signals which may span a broad temporal timeframe if sediment reworking has occurred. The ability to distinguish between local and distal sources of sedaDNA is of major importance in the interpretation of taxonomic profiles to reconstruct paleoenvironments.

Significance Statement Sedimentary ancient DNA (sedaDNA) has had a large impact on Quaternary science, revealing previously undetected and unexpected ecosystem profiles in the Pleistocene. Here, we show that the taphonomic origin of sedaDNA is evidenced in its association with sediment turnover, revealing that past environments can be classed as derived from a local, distal, or mixed input of sedaDNA. This approach is of fundamental importance in reconstructing past environments, shows a prevalence of mixed ecosystem signals that may require re-interpretation, and provides an avenue to the identification of reworked sedaDNA signals.

## Introduction

Over the past two decades sedimentary ancient DNA (sedaDNA) has revolutionized the resolving power of Quaternary science (1, 2), from the identification of a forested Greenland prior to the last glaciation (3) to more recently the recovery of unexpected ecosystem profiles over 2 million years ago (4). Correlates between environmental factors and DNA damage suggest that DNA may persist on the million-year time scale to a degree largely determined by temperature and temperature fluctuation (5), followed by other factors such as chemistry (6) and physical adsorption to protective mineral surfaces (7). However, concerns were raised early in the field about taphonomic processes, which could influence DNA deposition, leading to erroneous interpretations of sedaDNA (8).

Firstly, there are a number of taphonomic factors when interpreting sedaDNA profiles that should be considered. Among these, there may be biases in the amount of DNA deposited into the environment as a consequence of organism size (2) and genome size (9), leading to over- or underrepresentation of species. Secondly, postdeposition movement of DNA through potential diffusion (10), leaching (8), or sediment reworking (11) can lead to anachronous signals. Thirdly, considerations are required concerning the source of DNA deposited in a sediment (12). Sediments are usually laid down under fluids originating from a source environment, which may be some distance from the deposition environment and may well not reflect the local environment around the site of deposition. It is possible that sedaDNA derives from such incoming sediment, effectively representing a case of reworking or a general catchment signal, or alternatively, it may originate from the local environment being laid down concurrently with incoming sediment. In these scenarios, a sedaDNA signal could be reflective of a local, distal, or regional signal, leading to different sets of interpretations. Several studies have reported an apparently local source of sedaDNA (13–16), while there have also been recent attempts to build taphonomy models that incorporate the influence of catchment signals resulting from sedaDNA being brought with influxing sediments from surrounding regions (12); however, there remains no formal model that can be universally applied. There is therefore a need to integrate sedimentological data with sedaDNA data to better understand depositional sources of sedaDNA.

### A depositional framework integrating sediments and DNA

If the presence of sedaDNA is tightly linked to sediment types and sources, then the sedimentation regime should be highly influential on sedaDNA profiles. Here, we describe and test the robustness of a first-of-its-kind framework of a depositional model, developed using marine sediments (11). This framework measures the influence of sediment profiles on sedaDNA profiles, facilitating a measure of the influence of local and influxing sources of DNA on sedaDNA profiles.

**Figure 1:**
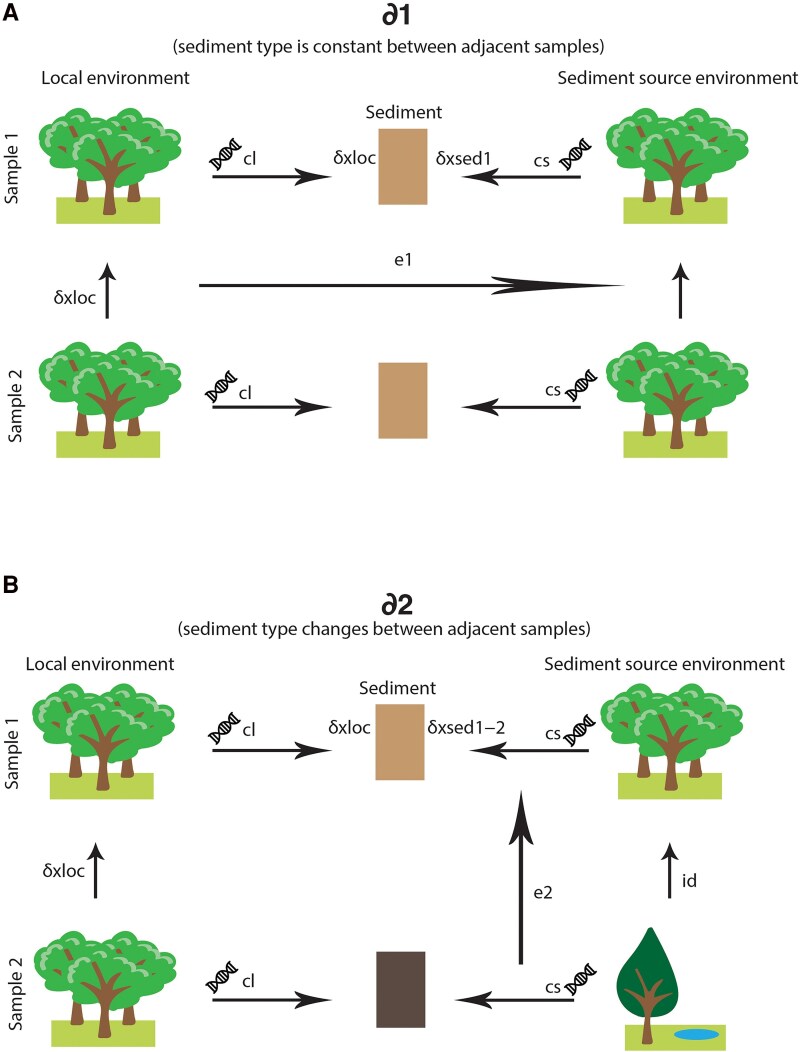
Sediment influx depositional model. A) ∂1 scenario: adjacent samples of same sediment type 1. B) ∂2 scenario: adjacent samples of sediment types 1 and 2. Parameters as follows: ∂1—ecological distance between adjacent samples of the same type; ∂2—ecological distance between adjacent samples of different sediment types; cl—proportion of sedaDNA contribution from local sources; cs—proportion sedaDNA contribution from influxing sediment from source environment; *δ*_loc_—change in ecological profile of local environment over time; *δ_x_*_loc_—contribution of *δ*_loc_ to ecological distance ∂ between samples; *δ*_sed1_—change in ecological profile of influxing sediment source environment over time; *δ_x_*_sed1_ contribution of *δ*_sed1_ to plant ecological distance ∂ between samples; id—ecological distance between different influxing sediment source environments 1 and 2; *δ_x_*_sed1-2_—contribution of id to ecological distance ∂ between samples; *e*_1_—environmental variable 1 describing the difference in rate of ecological profile change over time between δloc and δsed1; *e*_2_—environmental variable 2 describing the difference in influx rates from source environments 1 and 2.

In a depositional environment, when the type of sediment being deposited changes, it likely reflects some change in the source environment from which the sediment originates. This could be a physical change in locality from which the sediment originates or some other large change in that source environment that also could be associated with biotic turnover. If sedaDNA is directly associated with sediment also originating from the source environment, then sedaDNA profiles should be heavily influenced by sediment types and sources. However, if sedaDNA is predominantly from local sources independent of the sediment, then the sedimentation regime may be expected to be less influential on sedaDNA profiles. Recently, a framework integrating sedaDNA profiles and sedimentological data was developed on the basis of these assumptions and applied to marine core samples from the Doggerland area (11) (Fig. 1). In this model, the amount of biotic change between two time points is considered under two different sedimentation scenarios. The change in taxonomic profile, as evidenced in sedaDNA from one point in time to the next, is assumed to be a function of the amount of taxonomic change that has occurred in the local and the distal source environments, respectively, and their relative contributions to the sedaDNA profile. By considering two scenarios, one in which the sediment type, and by inference the source, has remained constant (distance value ∂1), and a second in which there is a change in sediment type (distance value ∂2), several model parameters are fitted, which explain the levels of local and distal contributions to the sedaDNA profile. Parameter fitting is achieved through a Monte Carlo approach to find values of parameters producing ∂1 and ∂2 that most closely match the observed measurements (see Methods).

This approach demonstrated that in a riparian (alluvial) system, fine silt or clay sediments, indicating slow flow rates, were associated with high influence of local contribution and low levels of influxing sedaDNA from distal sources (11). Conversely, coarse sand or gravel sediments, indicating more rapid flow rates, were associated with a high influence of influxing sedaDNA and a tendency toward mixed ecosystem and sediment reworking signals. Intuitively, such results make sense given the short half-life of DNA in water (6), indicating that sedaDNA does not persist in significant amounts relative to local DNA sources in slow moving systems.

However, this framework assumes that changes in sediment type are not generally correlated with wider climatic changes and regional biome turnover. In such a case, changes in sediment regime could be associated with variations in taxonomic profile both locally and distally, thereby causing a spurious correlation between sedimentation regime and taxonomic change. The sediment influx depositional model also does not take account of the possibility that different sediments may influence sedaDNA profiles through variant adsorbent properties (7), which could cause DNA associated with particular sediment types to become over or underrepresented. Furthermore, the general applicability of the framework is unclear since it has not been applied to nonriparian systems, such as lacustrine and terrestrial systems. Here, we test the robustness of the underlying assumptions of the framework and assess its general applicability to sedaDNA studies.

## Results

### Environmental associations of sediment regime and sedaDNA damage

To test the underlying assumptions of the depositional model framework shown in Fig. 1, we utilized data generated from Doggerland (17), as this represents the most comprehensive assembly of sediment types and associated sedaDNA profiles to date, and spans a timeframe from the Pleniglacial to the late Holocene (>16–4 kya) encompassing several major climatic shifts (Table S1). We used the subset of the data attributed to Viridiplantae from which ecological profiles can be built based on plant communities more easily than organisms from the other kingdoms. In that study, sediments were visually categorized and logged on the basis of sediment type (sands, gravels, silts, clays, peats, and chalks) and level of structure apparent through laminations (11). This is also the dataset on which the framework was originally applied, allowing us here to further interrogate the original model application and inferences.

**Figure 2:**
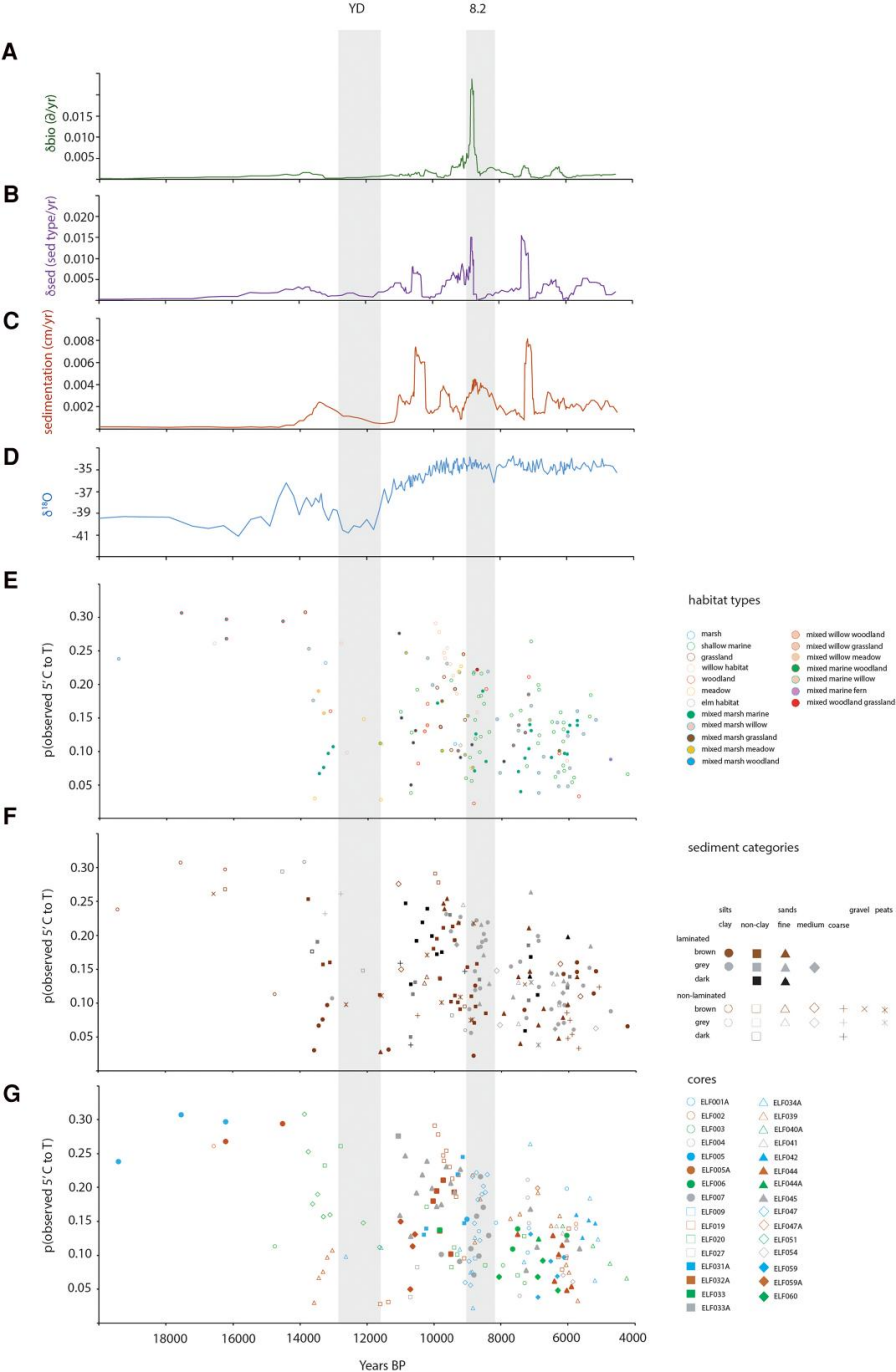
Environmental associations between sediment and sedaDNA. A) Average rate of change in distance (∂) values of taxonomic profiles between sequential sampling points in cores over time (δbio). B) Average rate of change in sediment category between sequential sampling points in cores (δsed). C) Average rate of accumulation of sediment between sequential sampling points in cores. D) Average δ^18^O values from GISP2 (17) corresponding to sampling points in cores over time. Graphs A–D) All based on sliding window average based on 10 chronologically sequential values across cores. Graphs E–G) Observed C to T mismatch frequencies at 5′ position 0 in DNA reads of individual samples categorized by habitat (E), sediment type (F), and core of sample origin (G). Younger Dryas (YD) and the 8.2 ka event (8.2) are marked by gray boxes.

Firstly, we considered whether rate of sedimentation changes (δsed, the number of changes in sediment category per year across cores, see Methods) correlated with major climatic shifts (Fig. 2). While δsed weakly correlates with the sedimentation rate (*R*^2^ = 0.1653, *P* = 1.98 × 10^−11^), neither δsed nor the sedimentation rate correlates closely with the δ^18^O profile from the GISP 2 ice core (18), reflecting Pleniglacial to Holocene temperature change (*R*^2^ = 0.0675 and 0.1174, *P* = 1.99 × 10^−5^ and 1.99 × 10^−8^, respectively). Both δsed and the sedimentation rate increase during the Bølling–Allerød interstadial (14.7–12.9 ka), rise episodically during the Pleistocene–Holocene boundary period between 11 and 9 ka and again between 7.2 and 7 ka, likely reflecting the time of marine inundation in this area (11). While there is clearly influence of major climatic shifts on δsed, the profile appears to be dominated by more local processes which are not reflected in the δ^18^O profile.

The biotic profile δbio (the rate of change of ∂ per year across cores) shows some increase during the Bølling–Allerød interstadial and at the Pleistocene–Holocene boundary but shows the largest change abruptly over a short period within the 8.2 ka cooling event, which was characterized by a period of rapid cooling from 9 to 8.2 ka (19). The data suggest a profound change in the Doggerland area during this period and a general profile of taxonomic change associated with broad climatic trends. The evident spike in both δbio and δsed rates around the 8.2 ka event indicates both that (i) sediments were turning over rapidly and (ii) ecology was in a state of flux. These two rates of change are highly correlated in this brief 84 year period (*R*^2^ = 0.6645, *P* = 6.17 × 10^−4^). Consequently, there is a considerable correlation between the two over the whole time series (*R*^2^ = 0.3695, *P* < 1 × 10^−15^). However, exclusion of this short period leads to a much lower correlation overall (*R*^2^ = 0.1336, *P* = 4.15 × 10^−9^). Furthermore, in this dataset, 13.9% of core samples were identified as influenced by DNA influxing with sediments, which could influence the overall correlation between δbio and δsed. Removal of this portion of the dataset marginally reduces the correlation (*R*^2^ = 0.1154, *P* = 2.26 × 10^−6^).

The underlying assumption of the sedaDNA influx model that changes in sediment type over time are not associated with general climate change is broadly satisfied, with ongoing local processes appearing to dominate. However, there is a low correlation between the rate of biotic change and rate of sediment change implying that both are subject to a level influence from local geomorphological processes in this dataset.

The sedaDNA influx model is based on determining the level of correlation between biotic change and sediment change to determine the extent to which incoming sediments are associated with sedaDNA. The natural low correlation observed may bias the model toward inferences of influxing DNA from distal sources rather than local deposition. Despite this potential bias, the majority of Doggerland sediments were identified as ones in which local deposition dominated, which suggests the model is robust to this level of background natural correlation.

We assessed the influence of sediment type on sedaDNA profiles. If DNA is adsorbed to surfaces of minerals in sediments, then its availability to hydrolytic attack should be reduced (7, 20–23). Expected consequences of this include a higher retention of DNA, greater persistence of DNA and a lower DNA damage profile associated with adsorbing sediments (24). We examined the extent of DNA 5′ cytosine deamination at the terminal most position of DNA molecules, causing C to T mismatches, as a measure of damage in sedaDNA (25) using MetaDamage (9; Fig. 2). Across all sediment types, there is a low but significant correlation between age and cytosine deamination (*R*^2^ = 0.1422, *P* = 3.78 × 10^−7^; Table S2). We considered individual types and category groups of sediments and found only three marginally significant correlations for laminated dark silts (*n* = 11), unstructured silts (*n* = 16), and nonclay laminated silts (*n* = 43). Broadly, the rate of cytosine deamination of silts matches the overall rate for all sediments at around 1 × 10^−7^ C to T %/year.

We further considered whether habitat type could significantly influence deamination rates, reflecting underlying factors such as salt concentration or pH (Table 1). Chemostratigraphic analysis has previously shown in this dataset that marsh environments are associated with high Br/Ti ratios indicative of elevated salinity (30). Examining habitats defined by taxonomic profile, we found three significant correlations for mixed woodland marsh (*n* = 9), mixed shallow marine willow (*n* = 21), and nonmarine marsh (*n* = 34) categories. In these cases, deamination rates were also close to the overall rate for all samples. Similarly, we found no clear instances of different levels of cytosine deamination between habitat types at different periods of time (Fig. 2). We considered whether the combination of habitat and sediment type may yield stronger correlations with cytosine deamination, but no convincing trends were revealed (Table 1).

**Table 1:** Influx signal (*cs*) estimates of published sedaDNA studies.

Sediment type^a^	∂1	∂2	N1^b^	N2	Environment	Estimated influx signal	Reference
Laminated olive green gyttja	0.01798511	0.8962228	19	6	lacustrine	0.8–0.9	Alsos et al (26)
Laminated rusty brown gyttja	1.00049571	0.38510625	7	4	lacustrine	0.7–0.8	Alsos et al (26)
Coarse detritus gyttja	0.00619743	0.00519	6	1	lacustrine	0.9–1	Alsos et al (26)
Laminated gyttja	0.0732458	0.066206	5	2	lacustrine	0.9–1	Alsos et al (26)
Laminated silty gyttja	0.0267445	0.3506005	2	2	lacustrine	0.8–0.9	Alsos et al (26)
Dark olive brown gytjja	0.0401616	0.4192	5	1	lacustrine	0.8–0.9	Alsos et al (26)
Olive brown silty gyttja	1.03317429	0.2096	7	2	lacustrine	0.7–0.8	Alsos et al (26)
Moss layer	0.2777775	0.5357135	2	2	lacustrine/terrestrial incursion	0.3–0.4	Alsos et al (26)
Banded white/gray silt	1.285	0.8026145	3	2	lacustrine/terrestrial incursion	0–0.1	Alsos et al (26)
UR1A: silty clay, laminated	0.122522	0.056553	10	2	lacustrine	0.9–1	Clarke et al. (27)
UR1B: organic-rich silty clay, medium coarse sand banding	0.08881287	0.1357835	62	2	lacustrine	0.9–1	Clarke et al. (27)
UR1C: mid-to-dark brown silty clay gyttja	0.0145835	0.215014	2	2	lacustrine	0.8–0.9	Clarke et al. (27)
Silt, organic remains	0.5	0.3	3	2	permafrost	0.3–0.4	Jorgensen et al. (14)
Fine sand	0.3666665	0.5	2	2	permafrost	0.2–0.3	Jorgensen et al. (14)
Silt	0.33484833	0.3392855	2	2	permafrost	0.3–0.4	Jorgensen et al. (14)
Laminated gyttja	0.7136	1.25	25	2	lacustrine	0.5–0.6	Pedersen et al. (15)
Sandy gyttja	0.05158775	0.048062	4	2	lacustrine	0.9–1	Pedersen et al. (28)
Laminated silt	0.07514689	0.048062	9	2	lacustrine	0.9–1	Pedersen et al. (28)
Lower clay silt	0.2038515	0.0157145	4	2	permafrost	0.6–0.7	Kjaer et al. (4)
Higher clay silt	0.058595	0.0157145	1	2	permafrost	0.7–0.8	Kjaer et al. (4)
Yellow brown clay silt	0.1552356	0.38966775	15	2	terrestrial	0.09^c^	Hansford (29)
Brown clay silt	0.777232	0.04067026	7	2	terrestrial	0.93^c^	Hansford (29)
Pale yellow brown clay silt	0.0416395	0.077269	2	2	terrestrial	0.09^c^	Hansford (29)
Red brown clay silt	0.17221418	0.0651155	12	2	terrestrial	0.76^c^	Hansford (29)
Pale brown clay silt	0.224106	0.01534254	2	2	terrestrial	0.52^c^	Hansford (29)
Silts	0.19085395	0.23986324	47	2	alluvial	0.03^c^	Allaby (17)
Sands	0.25161588	0.34178475	26	2	alluvial	0.05^c^	Allaby (17)
Clay silts	0.1735089	0.18124622	25	2	alluvial	0.05^c^	Allaby (17)
Nonclay silts	0.21056424	0.28946226	22	2	alluvial	0.11^c^	Allaby (17)
Laminated silts	0.19566623	0.26020191	40	2	alluvial	0.02^c^	Allaby (17)
Unstructured silts	0.08257781	0.1381699	2	2	alluvial	0.03^c^	Allaby (17)
Fine sand	0.15572061	0.20565963	17	2	alluvial	0.02^c^	Allaby (17)
Medium sand	0.19268852	0.59959364	4	2	alluvial	0.15^c^	Allaby (17)
Coarse sand	1.18329519	0.49666208	4	2	alluvial	0.7^c^	Allaby (17)
Laminated sand	0.12750505	0.33033684	16	2	alluvial	0.17^c^	Allaby (17)
Unstructured sands	0.47319382	0.36982104	10	2	alluvial	0.66^c^	Allaby (17)
Fine sands (unstructured)	0.32225045	0.22313632	4	2	alluvial	0.63^c^	Allaby (17)
Medium and coarse sands (unstructured)	0.57382274	0.47459584	6	2	alluvial	0.67^c^	Allaby (17)

^a^Sediment types follow categorizations of published studies.

^b^Number data points contributing to ∂1 (N1) and ∂2 (N2) values.

^c^cs estimated directly by Monte Carlo.

Finally, we considered whether the specific environments of individual cores influenced cytosine deamination (Fig. 2). We found a higher proportion of samples formed significant correlations (36%) when arranged by core rather than by sediment or habitat type, with significant correlations were found for ELF020 (*n* = 10), ELF045 (*n* = 18), ELF047 (*n* = 12), and ELF051 (*n* = 8; Table 1). However, we found little evidence for differences between cores. While Holocene aged samples show general overlap between cores, there is a single instance of significant difference in Bølling–Allerød aged samples between cores ELF039 and ELF051 (*P* = 0.0005626). In this case, the ELF039 samples have surprisingly low levels of cytosine deamination, lower than younger samples from the same core perhaps reflecting environmental differences at the time of deposition within the lake feature ELF039 is situated. We further considered specific sediment categories within specific cores but found no significant correlations (Table S2).

In summary, we find no strong evidence that sediments or habitats significantly differ in their rate of cytosine deamination that could be attributable to sediment-binding properties or underlying chemistry. Overall, individual cores with mixed sediment types provided the most significant correlations, suggesting the immediate core environment may be the most influential factor on the extent of cytosine deamination. Similar to our findings, previously cytosine deamination has been broadly correlated with age at a rate largely determined by regional environmental conditions of temperature and temperature fluctuation, but with high stochasticity over shorter time periods (5). If there is any contribution to DNA preservation through adsorption differences, it is not of significant effect in this dataset. It may be the case that larger datasets in the future may have the power to resolve such nuances. Overall, we detect no trends that would violate the underlying assumptions of the depositional model in this dataset.

**Figure 3:**
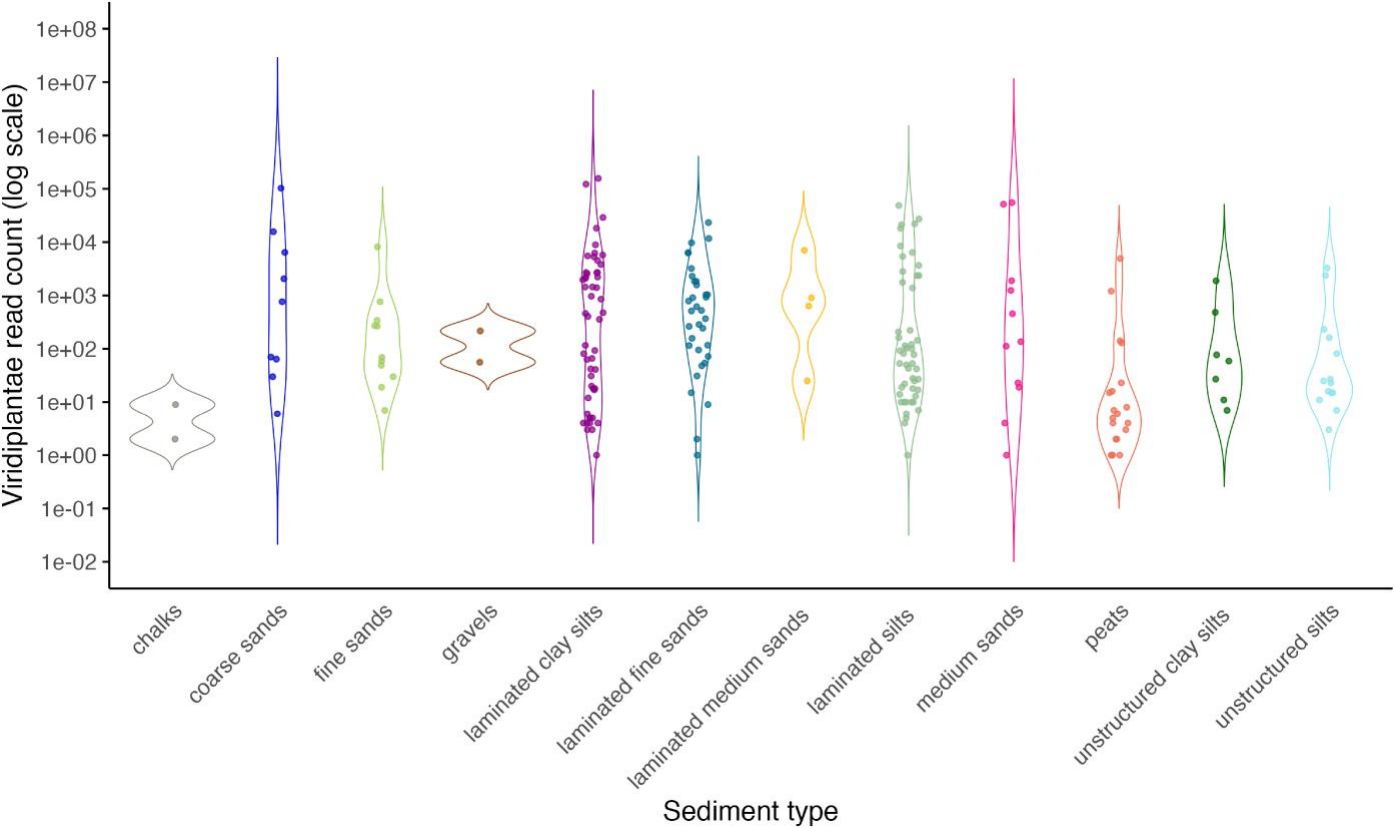
Read count of Viridiplantae in core samples by sediment type.

Finally, we surveyed total frequencies of “Viridiplantae”-assigned reads in associated sediment types. Unstructured sand sediments (mean Viridiplantae read count *µ* = 8,446, sediment sample number *n* = 32) yield on average an order of magnitude more reads than unstructured silts (*µ* = 418, *n* = 21) in this dataset, although the variance is generally wide between samples (Fig. 3). It may therefore be the case that an adsorbed sedaDNA fraction has remained adsorbed in the silts explaining the tendency to lower read counts recovered. Laminated sediments of all types are associated with high read counts (sands *µ* = 2,234 *n* = 36, silts *µ* = 5,619 *n* = 101), suggesting that laminations, often comprised of organic material, represent rich sources of sedaDNA. The differential recovery of DNA between sediments likely to adsorb DNA and those that are not, combined with no significant difference in base modifications supports the notion that sedaDNA profiles in this dataset are largely based on free DNA without a detectable contribution from DNA liberated from adsorption.

### Elucidation of an influx landscape based on sedimentation regime

**Figure 4:**
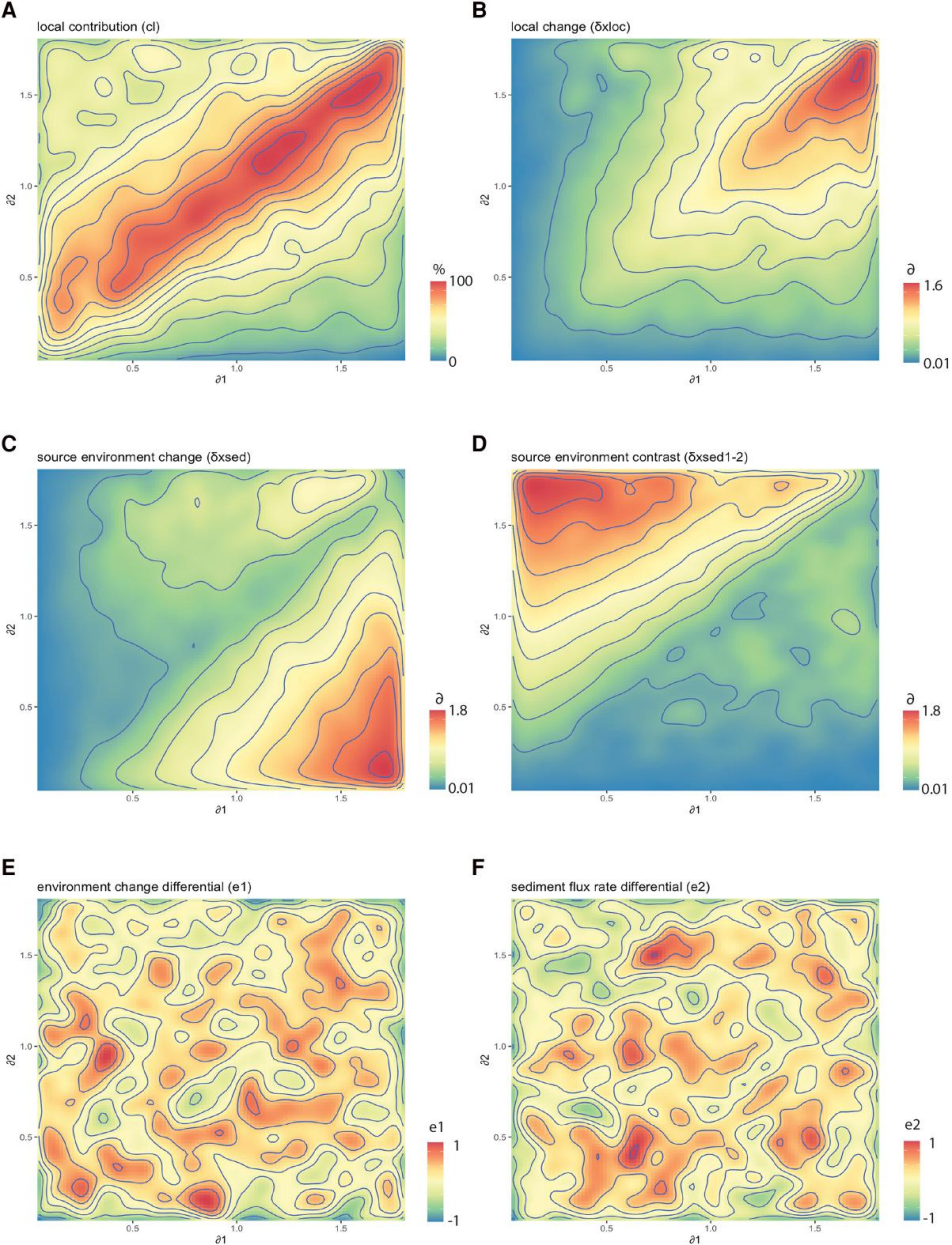
Parameter landscapes for sediment influx depositional model. Monte Carlo–based solutions (see Methods) for model parameters A) *cl*, B) *δ_x_*_loc_, C) *δ_x_*_sed_, D) *δ_x_*_sed1-2_, E) *e*_1_, and F) *e*_2_ for combinations of ∂1 and ∂2.

The sediment influx depositional model consists of eight parameters, cl (local contribution of sedaDNA), cs (contribution of influxing sedaDNA from distal source), *δ_x_*_loc_ (amount of change observed due to local environmental change), *δ_x_*_sed1_ (amount of change observed due to distal environmental change), *δ_x_*_sed1-2_ (amount of change observed due to change in distal source), id (difference in distal environments associated with sedimentation change), *e*_1_ (difference in extent of change of local relative to distal environments), and *e*_2_ (change in contribution of influxing sources associated with change in sedimentation regime due to factors such as dynamic flow rates; Fig. 1). Together these parameters contribute to the difference observed in taxonomic profile when sediment regime remains unchanged (∂1) and when sedimentation regime changes (∂2; see Methods). In order to understand how local and influxing sources of DNA are expected to change with varying values of ∂1 and ∂2 in this depositional model, we estimated parameters for six variables (cl, *δ_x_*_loc_, *δ_x_*_sed_, *δ_x_*_sed1-2_, *e*_1_, and *e*_2_) for given values of ∂1 and ∂2 to produce parameter landscapes (Fig. 4).

The landscape for local contribution (cl) shows that a high influence of local contribution is associated with situations in which the difference observed between taxonomic profiles is similar regardless of the sedimentation regime (Fig. 4A). Intuitively, when both ∂1 and ∂2 are high, it is associated with a greater level of taxonomic change in the local environment (Fig. 4B). However, a greater influence associated with influxing sediments is inferred when ∂1 and ∂2 become unbalanced, suggesting that changes to the sedimentation regime are influential on taxonomic profiles under these circumstances. When ∂1 is high relative to ∂2, it is associated with a greater level of taxonomic change in the distal environment from which the sediment is sourced. Conversely, when ∂2 is high relative to ∂1, it is associated with large changes in taxonomic profiles between changing sediment types, possibly suggesting likely changes in source location. These results suggest that even if sedaDNA may be inferred to have come from influxing sources and so not be appropriate for highly local interpretations, interpretations about the distal environment may be possible.

The two environmental variables, *e*_1_ and *e*_2_, do not produce highly structured landscapes and so do not appear to be broadly informative. These variables account for the fact that the rate of biological change, in terms of taxa turnover, may vary between local and distal environments (*e*_1_) or that the contribution of influxing DNA may change with changing flow rates over time between distal source and local environments (*e*_2_). Neither of these variables leads to a systematic explanation of varying ∂1 and ∂2 values, even though they may themselves vary.

### Application of the taphonomy framework to different depositional systems

While the depositional model framework appears validated on the extensive alluvial dataset from Doggerland (11), it has yet to be applied to different ecological systems in which the taphonomic regime is expected to differ. The parameter landscapes shown in Fig. 4 provide a look-up reference for any given values of ∂1 and ∂2, negating the requirement of a new Monte Carlo analysis to retrieve model parameter estimates. We leveraged this to examine published datasets of lacustrine (15, 26–28), permafrost (4, 14), and terrestrial (29, 31) systems from various dataset types and taxonomic profiling approaches in which there are sufficient sedimentological data to apply the depositional model (Table 1). Published datasets included both shotgun metagenomic and metabarcoding DNA sequence data and individual taxa and grouped taxa systems. For each study, we calculated values of ∂1 and ∂2 for different sediment types (see Methods), and used these to plot coordinates in the cl ∂1/∂2 landscape (Fig. 4A) to give estimates of distal source contribution to sedaDNA profiles (Table 1). Despite the relatively low sample number on which ∂ values were based, in many cases some informative trends emerged.

We examined 15 different sediment types of lake sediments across four studies (15, 26–28) (see Supporting Data S1–S4). In all but three cases, the ∂1/∂2 values were consistent with the majority of sedaDNA influxed from nonlocal regions in the depositional model, supporting previous findings of a regional sedaDNA signal from the catchment area for lake systems (12). Two exceptions are sequential samples taken from a shallow area of the glacial lake Andøya in Norway (3 m depth), dated to 14.18–12.73 ka (26). The strata were exceptional in the profile because of the occurrence of a moss layer and underlying silts consistent with erosion of acidic mire from the lake edge (26), which is reflected in the depositional model as a switch to local sources dominating the sedaDNA profile. The third example is from samples taken from Lake Comarum, a small (58 × 85 m) shallow (>1 m) lake in southern Greenland (15). The depositional model indicates an equable contribution to the sedaDNA profile from local and distal sources. Given the very small size of this lake it is not surprising that local vegetation in the immediate vicinity would have had a large influence on the sedaDNA profile leading to a mixed signal of local and distal sources.

We examined two permafrost studies. The first of these was based on three cores taken from around the Lake Tamyr area and three sediment types (14) (see Supporting Data S5). In this case, the depositional model solution indicates a domination of the sedaDNA signal from local sources, but an appreciable and consistent (25–35%) input from nonlocal sources, indicating the possibility of a mixed ecosystem signal. In this case, all cores were taken close to the lake edges, and while one might expect terrestrial systems to be dominated by a local signal in the absence of a strong external source of influx it is possible that periodic flooding could have introduced a more regional signal of sedaDNA over time. The second permafrost study was based on samples from Kap København formation in North Greenland (4) (see Supporting Data S6). We found one suitable sequence of samples for analysis from sample site 69 (stratigraphic Unit B2), where a change in the silt sediments is apparent with a contrast in clay content indicating some change in sedimentation regime. In this case, we estimate between 60 and 80% of the sedaDNA signal comes from distal sources leading to a likely mixed ecosystem signal, which matches the known taphonomy of the site of a terrestrial system washed into an estuary during an Early Pleistocene glacial minimum (32).

While lacustrine and permafrost systems are usually assumed to have little postdepositional DNA movement resulting from processes such as leaching (33), terrestrial systems often show signs of postdepositional DNA movement (8, 34). To mitigate this confounding issue, we examined terrestrial samples in which sedaDNA profiles had been tested for evidence of postdepositional movement (see Supporting Data S7). In this case, the level of statistical independence between sampling points was used to explore whether adjacent samples were significantly different or read counts of one sample could be explained by diffusion from the other (11), and no significant evidence of postdepositional movement was detected (29). These samples also show a stable chemostrigraphy that is consistent with no leaching (31). Samples were retrieved from the lower sections of Neolithic large man-made pits surrounding the settlement of Durrington Walls, below any instances of modern disturbance. In this case, sediment profiles are all silts, which are graded by color from yellow to red to brown. Yellow silts are consistent with a dominant local signal in the model. However, an increasing influence of distal sources is clearly correlated with progressively darker silt sediments, which for the most part overlay the lighter silts below. The current interpretation of chemostratigraphy and optically stimulated luminescence profiles at this site is that the pits may have been infilled manually at later points in history after the Neolithic (31). The depositional model suggests that this infilling was probably highly influential on the subsequent sedaDNA profile, and the sedaDNA itself appears to be highly associated with the source of coloration of the silts.

## Discussion

**Figure 5:**
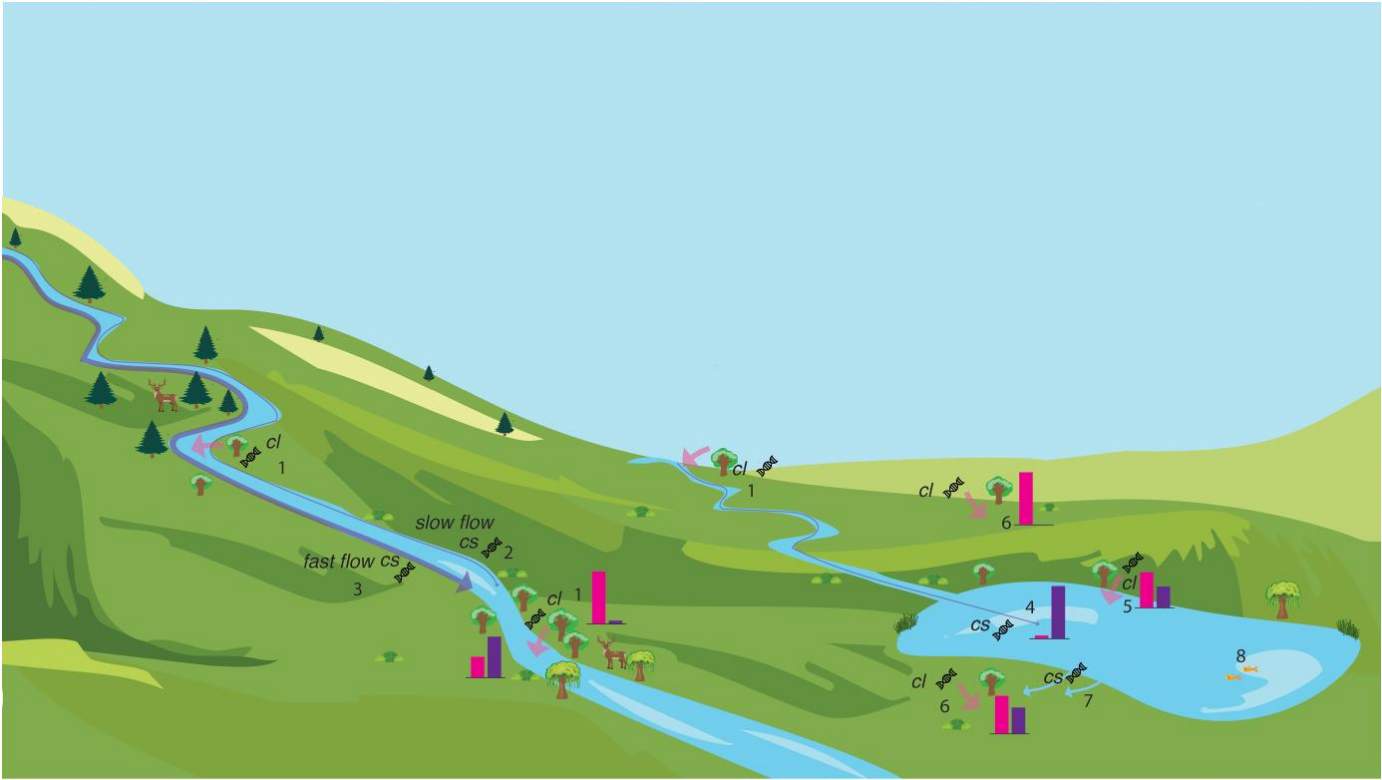
DNA deposition patterns based on sediment influx depositional model outputs from observed sedaDNA data. Arrows show direction of DNA movement, and arrow thickness represents quantity of DNA. Bar charts indicate relative contribution of local (pink) and distal (purple) sedaDNA sources. (1) Local contribution (cl) substantial in alluvial systems. (2) Little DNA is influxed (cs) in slow moving water due to DNA removal processes. (3) Substantial DNA can be influxed in rapidly moving water. (4) Lacustrine systems mostly receive influxed DNA with few local sources of DNA resulting in a dominant influx signal, even if little DNA is involved. (5) Lake margins can supply substantial local source DNA in the immediate vicinity. (6) Terrestrial systems are expected to be dominated by local sources in the absence of an influx source. (7) Events such as flooding can provide an influx source of DNA. (8) Faunal sources in lacustrine systems could potentially provide a dominating local source of DNA.

The depositional model is informative because of the dual nature of instances in which two critical variables are estimated, cl and *δ_x_*_loc_. While in the ∂1 scenario, independent variables cl, *δ_x_*_loc_, and *e*_1_ are influential; in the alternative ∂2, it is independent variables cl, *δ_x_*_loc_, *id*, and *e*_2_ which are influential. Therefore, solutions of cl and *δ_x_*_loc_ must satisfy both scenarios; consequently, two independent sources of information are consulted analogous to simultaneous equations. The relative simplicity of the model facilitates its applicability to a wide range of environments without the overfitting effects of further peripheral parameters. As such, the model appears to reveal meaningful trends with even quite small sample sizes given our survey of published studies here, but consideration should be given to the robustness of ∂1 and ∂2 measurements. The obvious further parameters that might be considered include the effects of various different sediment properties with regard to DNA adsorption, although we find no evidence that this has a confounding effect in our test dataset from Doggerland (11). It may be the case that sedaDNA datasets are derived largely from unadsorbed, free DNA, although further investigation into the extent of deamination in different sediment types may refine understanding. Kjaer et al. (4) observed differential recovery of DNA from model minerals with as little as 4% being recovered from the smectite-associated fraction of sediments, but 43% is recoverable from quartz, suggesting differential mineralogical make up of sediments could result in the differential read count we observe between sandy and clay rich deposits. If DNA were released from a state of adsorption, the expected effect would be to influence the amount and level of damage of DNA present rather than the taxonomic profile as a whole. While low-abundance taxa may be revealed by increased recovery from adsorbed DNA, they are not likely to be influential on overall taxonomic distance between samples. A more serious assumption of the model is the independence of sediment turnover and taxonomic turnover, that sediment changes do not necessarily reflect large events that also cause changes in taxonomic composition giving a spurious correlation through a common underlying variable. While it is unquestionably the case that some sediments reflect climatic conditions such as the loess formed under periglacial conditions (35, 36), on a local scale sediment turnover in our test dataset is driven by more frequent ongoing local processes with no clear general correlation to larger climatic events such as the Younger Dryas or Pleistocene–Holocene boundary. This local dominance of turnover suggests that the geomorphological processes that the model tracks through sediment transitions are not expected to be closely linked to taxonomic profile turnover. We did detect a low correlation between sediment transition and taxonomic profile turnover which could reduce the power to detect local contribution sources, although the sedaDNA influx model appeared to be robust in this case. This may not be the case for all studies, so it should be routinely considered. Furthermore, studies should consider whether there is sufficient temporal sampling in order to establish whether such a correlation exists or not. In sum, the sedaDNA sediment influx deposition model leads to a taphonomy framework illustrated in Fig. 5.

### Alluvial and lacustrine systems have contrasting sedaDNA taphonomies

The depositional model originally identified an association in alluvial systems of slow moving (silt and clay associated) systems with a dominance of locally sourced sedaDNA signal with little or no sedaDNA arriving with sediments (11). However, lake profiles of equably fine sediments show that sedaDNA originates from distal sources (Table 1). The half-life of DNA in fresh water is less than an hour in temperate ecosystems (6), which should seemingly apply in both taphonomic contexts, alluvial and lacustrine. From this, we conclude that the amount of sedaDNA arriving from distal sources is likely to be comparable between the two and the difference between the estimates of local versus distal contributions must be in the absolute amount of DNA coming from local sources. A corollary is that lacustrine sedaDNA profiles are typically expected to be based on a lower absolute amount of DNA than alluvial profiles. It is intuitive that lakes provide little direct plant DNA from local sources if they are too deep for aquatic plants. However, it may be the case that a different taphonomic profile may be obtained from animal DNA than plant DNA in lacustrine systems where substantial local contribution may be made by fish and aquatic invertebrates.

### The prevalence of mixed ecosystem signals

Where distal sources of DNA are dominantly influential on the sedaDNA profile such as in lacustrine sediments the signal is likely to reflect a regional catchment signal, which is likely to include a range of ecosystem types. Where there is a large input of DNA from the immediate vicinity relative to any influxing material, as is expected to be the case for terrestrial systems, slow moving narrow river systems, and very small bodies of water, the signal is likely to strongly reflect the immediate ecosystem. However, there are numerous instances shown here in which there is evidence of input from both local and distal sources, with neither completely dominating. In such instances, caution needs to be applied when making inferences about the local environment, or indeed the regional profile since the signal will be distorted by the type of local environment. It is therefore likely that many current published datasets represent mixed ecosystem signals, and some reappraisal may be appropriate. This is particularly the case for systems in which there has been past fast influxing flow, typically leading to medium to coarse sandy unstructured deposits. Where a large number of sedimentological shifts can be sampled in studies, more robust estimates of deposition sources will be possible.

### Sediment reworking signals can be revealed under some circumstances

Where sedaDNA profiles can be attributed to distal sources, it is possible that the DNA can reflect the contemporaneous distal environment or potentially reworked sedaDNA from an earlier time. If such reworked deposits are not vastly different in age, for example by only a few thousand years, it is unlikely that reworking would be discernible on the basis of clearly greater damage signatures in the older DNA given the stochasticity in cytosine deamination profiles, as apparent in Fig. 2. However, sedaDNA of significant age, such as over half a million years, is typically associated with high frequencies of observed C to T mismatches indicating saturation of signal (4, 37, 38) and so could be discernible from the unsaturated Holocene to late Pleistocene aged associated damage as outlined in Fig. 2. In such cases, it may be possible to infer a reworking signal. Alternatively, reworking may be inferred where a sedaDNA profile attributed to an influxing signal closely resembles an earlier likely source. This was the case in the Southern River system in which sandy deposits identified as an influxing signal in core ELF031 showed marshy grassland profile in contrast to underlying locally sourced deposits. However, upstream almost identical taxonomic profiles of local source were identified a few hundred years earlier in age providing convincing evidence of a reworking episode (11).

### Concluding remarks

The model has been applied here to a range of data types, metagenomic and metabarcoding, and taxonomic treatments categorizing at genus, family, or composite levels. The model is theoretically applicable for any internally consistent system for measuring taxonomic distance between samples, although it may be the case that different levels of taxonomic resolution may give differing levels of resolution of taphonomic profile. The sediment influx depositional model is therefore a potentially powerful tool to aid interpretation of sedaDNA profiles and highlights the need for comprehensive sedimentological data to be included in sedaDNA studies. In particular, the pervasive nature of mixed ecosystem signals combined with the current limits in identifying reworked signals underscores the caution required in interpretations and the need to be able to identify where profiles represent local or distal profiles.

## Methods

### Environmental correlation analysis

The environmental correlation analysis was carried using data from reference (11). All analyses were carried out using shotgun metagenomic sedaDNA samples. The rate of biotic change between adjacent samples in a core (δbio; Fig. 2) was calculated using the difference in ecological distances (∂ values) between two consecutive samples (Table S1), divided by the difference in age of the samples:


(1) $$\delta \mathrm{bio}=\frac{\partial }{t_{1}-t_{2}}.$$

To calculate the rate of sediment change (δsed; Fig. 2), Eq. 2 was used where *x* is the number of transitions between sediment types between two consecutive sampling points in a core, and *t*_1_ and *t*_2_ are the respective ages of the older and younger samples:


(2) $$\delta \mathrm{sed}=\;\frac{x}{t_{1}-t_{2}}.$$

The inferred rate of sedimentation was estimated from the difference in depth between sampling points in a core (Δ cm, Table S1) and the date of each sample point.

A sliding window average approach was used for both sediment and biotic rates of change based on 10 data points of δbio, δsed, and inferred sedimentation rate across cores with samples arranged in chronological order. The stable isotope profile was extracted from reference (18), with the closest δ^18^O value in age ascribed to each sample. The same sliding window approach was then used to generate the profile in Fig. 2.

The amount of cytosine deamination was determined using MetaDamage with samples where read counts exceed 300, which has previously been shown to be a sufficient sample size (9). Samples were organized in sediment, habitat, and core categories (Fig. 2). Sediment categories of samples followed the core logs of reference (11). Primary habitat types were determined using the dominant ecosystem profile type from reference (11) (Fig. 2, clear circles). In samples where two ecosystem profile types dominate, mixed categories were used which were combinations of the primary habitat types (Fig. 2, solid circles). In order to compare read counts between samples, we restricted the analysis to samples of equable sequencing effort found in Supplementary Information 2 in reference (11). Violin plots were generated in R (39) using total Viridiplantae read counts of samples, separated into their sediment categories (Supplementary Data S2 (11)).

### Basic model outline

The model outlined in Fig. 1 is based on differences in taxonomic profile (∂) that are measured as a difference in the sum of squares (dss) statistic of proportions of sedaDNA read profiles belonging to each taxonomic unit:


(3) $$\partial_{\mathrm{observed}}=\;\sum_{i=1}^{N}{\left(\;p_{1i}-p_{2i}\right)}^{2}.$$

Where there are *N* taxonomic units, *p*_1i_ and *p*_2i_ are the proportions of the *i*th taxonomic unit in samples 1 and 2, respectively. Observed values of ∂1 and ∂2 were estimated from the average of all observed instances of adjacent samples within the same unchanged sediment type (∂1), and adjacent samples spanning sediment transitions (∂2). There is a general assumption that the mean time difference between samples in these two scenarios does not significantly differ reflecting a relatively even sampling regime. This assumption is met in the test dataset used in this study for both distance (Δ cm) and time (Δ years) between the two categories (Table S1).

The model difference expected under ∂1 is expressed as the sum of the amount of change due local environmental change (*δ_x_*_loc)_ and amount of change due to distal environmental change associated with sedaDNA influxing with sediment (*δ_x_*_sed1_):


(4) $$\partial 1=\delta_{x\mathrm{loc}\;}+\delta_{x\mathrm{sed}1}.$$

Where the amount of change attributable to influxing sedaDNA is a function of the relative proportional contributions of *δ_x_*_loc_ (cl) and *δ_x_*_sed1_ (cs where cs = 1 − cl) and the rate of environmental change in the distal environment relative to the local environment (*e*_1_):


(5) $$\delta_{x\mathrm{sed}1}=\;\frac{\delta_{x\mathrm{loc}}}{\mathrm{cl}}\cdot \mathrm{cs}(1+e_{1}).$$

The model difference expected under ∂2 is expressed as the sum of the amount of change due local environmental change (*δ_x_*_loc_) and the amount of change due to and the change associated with the compositional difference between sediments 1 and 2 (*δ_x_*_sed1-2_):


(6) $$\partial 2=\delta_{x\mathrm{loc}\;}+\delta_{x\mathrm{sed}1\text{-}2}.$$

Where the amount of change attributable to influxing sedaDNA (*δ_x_*_sed1-2_) is a function of the change of the taxonomic profiles between sediments 1 and 2 (id), the relative proportional contribution of incoming sedaDNA (cs), and the associated change in that contribution associated with changing flow rates associated with the different sediment types (*e*_2_):


(7) $$\delta_{x\mathrm{sed}1\text{-}2}=\mathrm{id}\cdot \mathrm{cs}\cdot (1+e_{2}).$$

### Generation of parameter landscapes

In order to estimate parameter values from the model from observed data, we used a Monte Carlo approach. For each pair of values of *e*_1_ and *e*_2_, there are three independent variables cl, *δ_x_*_loc_, and id, from which all other values are derived. Monte Carlo chains began with random values of *e*_1_ and *e*_2_ in the range of −1 to +1, followed by random increments of 0.01, for which all values of cl, *δ_x_*_loc_, and id were explored in increments of 0.01 in the ranges of 0–1, 0–2, and 0–2, respectively. For each set of parameter values, model values of ∂1 and ∂2 were calculated using Eqs. 2–4, and the score of the fit to observed values of ∂1 and ∂2 as follows:


(8) $$\mathrm{fit}=\sqrt{{\left(\partial 1_{i}-\partial 1\right)}^{2}}+\sqrt{{\left(\partial 2_{i}-\partial 2\right)}^{2}}+\sqrt{{\left(\frac{\partial 2_{i}}{\partial 1_{i}}-\frac{\partial 2}{\partial 1}\right)}^{2}}.$$

Unheated chains were terminated after 20 attempts to improve fit had failed and chains were repeated 1,000 times. The entire parameter space is 1.6 × 10^11^, and chains were sufficient to explore most if not all this space.

In order to generate the landscapes parameters were estimated as described above for values of ∂1 and ∂2 ranging from 0.05 to 1.8 in 0.05 increments. Density plots with contours were generated using ggplot in R (40).

### Estimation of ∂1 and ∂2 values from published datasets

In the case of lacustrine (15, 26–28) and permafrost (4, 14) datasets, the taxonomic unit used to calculate ∂ values was the species. The unit in the case of the terrestrial (29, 31) and alluvial (11) systems was the guild unit, a group of species shown to co-occur in the data (see reference 11 for details). Authors’ measurements (read count or metabarcoding presence) were converted into proportions of all observations for each sample, from which dss statistics were calculated. All workings are shown in Supplementary Data S1–S5.

## Supplementary Material

pgag271_Supplementary_Data

## Data Availability

DNA sequence data used to validate the model have previously been deposited in NCBI SRA (PRJNA1001812) in reference 11. Code used for the Monte Carlo algorithm is available in the Supplementary material.

## Preprints

This manuscript was posted on a preprint at https://www.biorxiv.org/cgi/content/short/2025.04.30.651409v1.
