# Supplementary material for: A depositional taphonomy model for sedaDNA based on biotic profiles of contrasting sedimentation regimes
Source: PNAS Nexus. 2026 Aug 10;5(8):pgag271. doi: 10.1093/pnasnexus/pgag271 (PMC13484878; doi:10.1093/pnasnexus/pgag271)
Supplement: Supplementary file 1 [file pgag271_supplementary_data.zip › PNASNEXUS-PNASNEXUS-2026-00891-T-s04.rtf]

##############################################
# A Hill Climbing process to converge        #
# on the best solution of sediment and local #
# contributions to change.                   #
# Robin Allaby UoW 2021                      #
##############################################


$d1 = $ARGV[0];
$d2 = $ARGV[1];
#$ratio = $ARGV[2];
$ratio = $d2/$d1;
# initialize parameters:


		my $comploc = 0.01; my $dloc = 0.01; my $influxdiff = 0.01;
		my $complocbest = 0; my $dlocbest = 0; my $scorebest = 100;
		my $compsed = (); my $dsed = (); my $d1i = (); my $d2i = (); my $ratioi = ();
		my $i = 0.01; my $j = 0; my $k = 0; my $mcscore = 100;
		my $next = 0; my $it = 1; my $jump = 1;

	do {
		#open (OUT, ">brute_matrix") or die "Cannot write output matrix\n";
		my @dlocs = ();
		while ($influxdiff < 2) {
			$comploc = $i; $dloc = $i;
		while ($dloc < 2) {
			$comploc = $i; my @complocs = ();
			while ($comploc < 1) {
						$compsed = 1-$comploc;
						$dsed = ($dloc/$comploc)*($compsed*(1+$j));
						$d1i = $dloc + $dsed;
						$dsed12 = $influxdiff*($compsed*(1+$k));
						$d2i = $dloc + $dsed12;
						$ratioi = $d2i/$d1i;
						$score = sqrt(($d1-$d1i)*($d1-$d1i)) + sqrt(($d2-$d2i)*($d2-$d2i)) + sqrt(($ratio-$ratioi)*($ratio-$ratioi));
						push (@complocs, $score);
						if ($score < $scorebest) { $scorebest = $score; $dlocbest = $dloc; $complocbest = $comploc; $influxdiffbest = $influxdiff;}
						#$comploc = $comploc + $i;
						#print OUT "d1i: $d1i, d2i: $d2i, comploc: $comploc, compsed: $compsed, dloc: $dloc, dsed: $dsed, dsed12: $dsed12, ratioi = $ratioi, score: $score\n"; 
						$comploc = $comploc + $i;
			}
			push (@dlocs, @comploc);
			$dloc = $dloc + $i;

		}
			$influxdiff = $influxdiff + $i;
		}
		#close OUT;
 		$comploc = $complocbest;
		$dloc = $dlocbest;
		$score = $scorebest;
		$compsed = 1-$comploc;
		$influxdiff = $influxdiffbest;
                                                $dsed = ($dloc/$comploc)*($compsed*(1+$j));
						$dsed12 = $influxdiff*($compsed*(1+$k));
                                                $d1i = $dloc + $dsed;
                                                $d2i = $dloc + $dsed12;
                                                $ratioi = $d2i/$d1i;

	#	print "\n\nInput:d1:$d1\td2:$d2\tratio:$ratio\n";
	#		print "d1:$d1i\td2:$d2i\tdloc:$dloc\tdsed:$dsed\tdsed12:$dsed12\tcomploc:$comploc\tcompsed:$compsed\tinfluxdiff:$influxdiff\tratio:$ratioi\t";
	#	print "score:$score\n";

		# check whether we have an improvement, if so accept and try again
		if ($score < $mcscore) {
			$mcscore = $score; $mcd1i = $d1i; $mcd2i= $d2i; $mcdloc = $dloc; $mcdsed = $dsed; $mccomploc = $comploc;
			$mccompsed = $compsed; $mcinfluxdiff = $influxdiff; $mcratioi = $ratioi; $mcj = $j; $mck = $k; $mcdsed12 = $dsed12;
			$mccheck = 0;
		print "Iteration $it score $mcscore\n";

		} else {
			$mccheck++;
			if ($mccheck >20) {
				# no improvement in 20 iterations
				# then jump in j k parameter space
				$j = (rand(100))/100; $k = (rand(100))/100;
				$w = rand(1000); if ($w>500) {$w=1;} else {$w=-1;}
				$v= rand(1000);  if ($v>500) {$v=1;} else {$v=-1;}
				$j = $j*$w; $k = $k*$v;
				print "jump $jump\n"; $jump++;
				$mccheck = 0;
				if ($jump == 1000) {$next = 1;}
			}
		}	
		$x = rand(1000); $y = rand(1000);
		if ($y>500) {
				if ($x>500) { $j= $j+0.01;} else {$j = $j-0.01;} 
		} else {
				if ($x>500) { $k = $k+0.01;} else {$k = $k - 0.01};
		}
	$it++;

	} until ($next ==1);
		$s1change = (1+$j);
		$s2change = 1+$k;		

	        print "\n\nInput:d1:$d1\td2:$d2\tratio:$ratio\n";
                print "d1:$mcd1i\td2:$mcd2i\tdloc:$mcdloc\tdsed:$mcdsed\tdsed12:$mcdsed12\tcomploc:$mccomploc\tcompsed:$mccompsed\tinfluxdiff:$mcinfluxdiff\tratio:$mcratioi\n";
		print "source 1 change relative to local $s1change\nsource 2 presence relative to source 1 $s2change\n"; 
                print "score:$mcscore\n";
		open (OUT, ">sedout") or die "Cannot write sedout file\n";
		print OUT "$mccomploc\t$mccompsed\t$mcdloc\t$mcdsed\t$mcdsed12\t$mcinfluxdiff\t$s1change\t$s2change\t$d1\t$d2\t$mcscore\n";
		close OUT;
